# RELATIONSHIP BETWEEN THE DIET QUALITY INDEX IN NURSING MOTHERS AND THE FATTY ACID PROFILE OF MATURE BREAST MILK

**DOI:** 10.1590/1984-0462/2021/39/2019089

**Published:** 2020-07-03

**Authors:** Ronilson Ferreira Freitas, Mariana de Souza Macedo, Angelina do Carmo Lessa, Nísia Andrade Villela Dessimoni Pinto, Romero Alves Teixeira

**Affiliations:** a Universidade Estadual de Montes Claros, Montes Claros, MG, Brazil.; b Universidade Federal de Viçosa, Viçosa, MG, Brazil.; c Universidade Federal dos Vales do Jequitinhonha e Mucuri, Diamantina, MG, Brazil.

**Keywords:** Food consumption, Healthy diet, Maternal nutrition, Fatty acids, Human milk, Consumo alimentar, Dieta saudável, Nutrição materna, Ácidos graxos, Leite humano

## Abstract

**Objective:**

To evaluate the relationship between the maternal diet quality and the fatty acid composition of breast milk in the first trimester of lactation.

**Methods:**

This is an observational cross-sectional epidemiological study of nursing mothers. The data collection instruments were a semi-structured questionnaire for sample characterization and a recall of usual intake. Diet quality was assessed based on the healthy eating index (HEI). Samples of mature breast milk were obtained by hand milking. Milk fat was extracted using the Bligh-Dyer method and methylated with 0.25 mol/L sodium methoxide in methanol diethyl ether. A gas chromatograph equipped with a flame ionization detector determined the milk fatty acid profile. Pearson’s and Spearman’s correlation tests evaluated association between the variables. Subsequently, a multiple linear regression model was built and multivariate regression analysis was applied.

**Results:**

Our findings revealed an inverse relationship between the consumption of total fruits and the polyunsaturated fatty acid profile and a direct association of the intake of total fruits and total grains with monounsaturated and saturated fatty acids.

**Conclusions:**

The results of this study suggest that maternal diet quality affects the fatty acid composition of breast milk.

## RESUMO

**Objetivo:**

Avaliar a relação entre a qualidade da dieta materna e a composição do leite humano em ácidos graxos no primeiro trimestre de lactação.

**Métodos:**

Trata-se de um estudo epidemiológico observacional e transversal com nutrizes. Os instrumentos de coleta dos dados foram questionário semiestruturado para caracterização da amostra e o recordatório de ingestão habitual. A qualidade da dieta foi avaliada por meio do índice de alimentação saudável (IAS). Amostras de leite materno maduro foram obtidas por meio de ordenha manual. A extração da gordura do leite ocorreu mediante o método de Bligh-Dyer, e essa gordura foi metilada com metóxido de sódio 0,25 mol/L em metanol dietil éter. O perfil de ácidos graxos do leite foi determinado por um cromatógrafo a gás equipado com detector por ionização de chamas. Realizaram-se teste de correlação de Pearson e teste de Spearman para verificar associação entre as variáveis. Posteriormente se aplicaram o modelo de regressão linear múltiplo e a análise de regressão multivariada.

**Resultados:**

Foi possível observar relação inversa entre o consumo de frutas totais e o perfil de ácidos graxos poli-insaturados e relação direta entre o consumo de frutas totais e cereais totais e os ácidos monoinsaturados e saturados.

**Conclusões:**

Sugere-se que a qualidade da dieta reflete na composição de ácidos graxos do leite materno.

## INTRODUCTION

Breast milk is considered the appropriate and ideal food for infant nutrition, since it is rich in vitamins, proteins, carbohydrates, fats, minerals, and water. Thus, protecting, promoting, and supporting breastfeeding has become an important strategy in improving the health status of children.^1^

Among the constituents of breast milk, we highlight the lipids, composed of fatty acids (FA), which are important to the growth and development of infants in their first six months of life, in addition to ensuring their health. These FA contribute to the development of the brain, nervous system, and visual functions; prevent gastrointestinal, respiratory, and urinary tract infections; and have a protective effect against allergies.^2^ Studies have reported that the total lipid content and the FA composition of breast milk can vary, and factors such as the stage of lactation, maternal age, gestational age at birth, daily variation between lactations, genetic characteristics, nutritional status, and eating habits can influence the fat content and FA composition of breast milk.^3^ ^,^ ^4^ However, eating habits and maternal diet have proven to be the main modulation factors for the FA composition of breast milk,^3^ arousing the interest in evaluating the effect of the overall diet quality of nursing mothers on the composition of breast milk.

With the growing interest in assessing the overall diet quality of various population groups, many methods have been developed and adapted to these groups and used to analyze specific health outcomes.^5^ ^,^ ^6^ ^,^ ^7^ Among these methods, the healthy eating index (HEI), elaborated by Kennedy et al.^5^ for the United States Department of Agriculture, in 1995, aims to evaluate the overall diet quality, incorporating, in a single measure, the food and nutritional recommendations.

The composition of these indices has continuously been updated and reviewed, following the recommendations from food guides and specific nutritional guidelines, resulting in new indices adjusted for determined population groups.^8^ However, standardized instruments, such as HEI, adapted to nursing mothers and studies on food consumption in the postpartum period are rare in the literature. In Brazil, we found one study, conducted by Tavares et al.^9^ and published in 2013, that assessed the diet quality of nursing mothers using an adapted version of HEI.

In this context, studies that evaluate the lipid composition of breast milk and the lipid intake in nursing mothers using dietary surveys become relevant, given the scarcity of investigations on the association between the overall maternal diet quality and the composition of breast milk. Considering this premise, the present study aimed to evaluate the relationship between the maternal diet quality and the FA composition of breast milk in the first trimester of lactation.

## METHOD

This work is part of a research project entitled *Avaliação da relação entre estado nutricional de iodo materno durante a gestação e lactação e incidência da deficiência de iodo entre recém-nascidos e lactentes no município de Diamantina, Alto do Vale do Jequitinhonha, MG* (Evaluation of the relationship between the maternal iodine status during pregnancy and lactation and the incidence of iodine deficiency among newborns and infants in the city of Diamantina, Alto Vale do Jequitinhonha, Minas Gerais). This is an observational cross-sectional epidemiological study based on the FA composition of mature breast milk and the dietary assessment of nursing mothers enrolled in the Family Health Strategy (*Estratégia Saúde da Família* - ESF) in Diamantina (MG).

We defined the sample population by considering the total number of pregnant women registered and followed in each of the nine public health units of the municipality in two years: 264 and 282 in 2013 and 2014, respectively. Based on the arithmetic mean, the study population comprised 273 pregnant women. This research used samples of breast milk from 106 nursing mothers who participated in the larger study and who gave birth between August 2014 and December 2015, that is, the first 106 women included in the main study. The inclusion criteria were: single birth and living in the urban area. Mothers who had a prolonged hospitalization or diagnosed with diseases that required the interruption of breastfeeding were excluded from the study. Only nursing mothers who signed the informed consent form participated in the study.

A previously trained team collected the data by administering a recall of usual intake and a questionnaire that covered demographic characteristics (age, ethnicity, marital status, and maternal schooling), socioeconomic aspects (paid work, household income, and benefits), nutritional status (body mass index - BMI: pre-gestational and at the last prenatal visit), and obstetric history (gestational age). In addition to the questionnaire, a sample of mature breast milk was collected to evaluate the FA content. Colostrum and transitional milk were not analyzed. Pre-gestational BMI was classified according to the criteria defined by the World Health Organization (WHO)^10^ for adults: underweight (<18.5), normal weight (≥18.5 and <25), overweight (≥25 and <30), and obesity (≥30). The classification of the initial nutritional status of women into underweight, normal weight, overweight, and obesity considered the critical levels of BMI for the gestational age adopted by the Brazilian Ministry of Health.^11^

The definition of gestational age as preterm and term followed the International Statistical Classification of Diseases and Related Health Problems, which determines that preterm infants are those born with less than 37 weeks of gestational age and term infants are the ones born with at least 37 weeks of gestational age.^12^

Information relating to food consumption was gathered by a recall of usual intake based on a 24-hour recall, which had questions about the usual intake at each meal. Data collected in household measures were converted into grams or milliliters, thus enabling the detailed nutritional analysis of food intake. We analyzed the nutritional composition of the usual diet of each nursing mother with the Avanutri^®^ software, version 3.

The overall diet quality was assessed using the HEI proposed by Guenther et al.^13^ ^,^ ^14^ and adapted by Previdelli et al.^7^ This index consists of 12 components: nine food groups (total grains; whole grains; meat, eggs, and legumes; total fruits; whole fruits; total vegetables; dark green and orange vegetables and legumes; milk and dairy; oils), two nutrient-based components (saturated fat and sodium), and one element that corresponds to the total energy intake of solid fats (saturated and trans), alcohol, and added sugar (SoFAAS).^8^ The HEI construction involved evaluating the amount of food consumed declared in the recall, weighting them according to the daily intake recommended by the Dietary Guidelines for the Brazilian Population,^15^ adjusted for 1,000 Kcal.

Foods not listed in the food group servings of the Dietary Guidelines for the Brazilian Population^15^ were weighted according to the energy value of the serving. Foods predominantly composed of simple carbohydrates (soft drinks, hard candies, candies, gelatin, etc.) were classified into the group of sugars and candies.

For the HEI analysis, we separated the ingredients of culinary preparations with items from different food groups (cakes, pasta with sauce, meat dishes) and computed them in each corresponding group. Similarly, the components of processed foods with ingredients from different food groups (cookies and candies) were also isolated and standardized according to the label information.^16^

In the HEI construction, each component received a score based on the number of servings consumed per 1,000 Kcal (food groups), mg/1,000 Kcal (sodium), or proportion of the total energy intake per nutrient (SoFAAS). The maximum total HEI score was 100 points. The minimum score for individual components was zero, and maximum scores ranged between 5, 10, or 20, depending on the component.^7^ ^,^ ^13^ The score for intermediate intake values, comprising the interval between the minimum and maximum score criteria, was attributed proportionally.

The component meat, eggs, and legumes had its score estimated by the sum of the energy value of the meat, egg, and legume groups. When this group reached its maximum score, the energy value from legumes was computed in the groups dark green and orange vegetables and legumes and total vegetables, simultaneously.^7^

Regarding the total score, the closer to the maximum score, the better the diet quality. As the index was developed to represent different dietary aspects, no classification is adequate or inadequate considering the total score. Therefore, each component should have their score evaluated separately.^13^ ^,^ ^14^

The FA profile was analyzed based on samples of up to 15 mL of breast milk, collected by fasting nursing mothers using the hand milking technique, after the first breastfeeding of the morning, from the same breast they fed their child. The milk was stored at -80ºC until the analysis.

The milk FA profile was analyzed in the Chromatography Laboratory of the Universidade Federal de Minas Gerais. Fat was extracted from 0.8 mL aliquots of breast milk using the Bligh-Dyer method and methylated with 0.25 mol/L sodium methoxide in methanol diethyl ether (1:1).^17^

The analyses were carried out in an HP7820A gas chromatograph from Agilent Technologies (Wilmington, USA) equipped with a flame ionization detector - EZChrom Elite Compact data acquisition software (Agilent, Wilmington, USA). We used an SP2560 column 30 m × 0.25 mm × 0.20 µm (Supelco, Pine Hall Rd, USA) with a temperature gradient of 80ºC, 0 min, 7ºC/min up to 240ºC; injector (split of 1/30) at 250ºC and detector at 260ºC. Hydrogen was used as carrier gas (3.0 mL/min), and the injection volume was 1 µL. Peaks were identified by comparing patterns of FAME C14-C22 methylated FA (Supelco cat No. 18917, Pine Hall Road, USA).

Data analysis was performed in the software Statistical Package for Social Sciences (SPSS), version 20.0 (Chicago, USA). To that end, we estimated the mean percentages and standard deviations, medians, and interquartile ranges of total HEI, each of its components, and the FA present in breast milk, as well as the percentage of maximum and minimum scores. The Kolmogorov-Smirnov test assessed the normality of the data.

We used Pearson’s and Spearman’s correlation tests to check for association between the variables. The variables with p<0.20 in this analysis were included in the multiple linear regression model. Subsequently, we carried out a multivariate regression analysis, considering the HEI total and component scores as the independent variables and the different FA contents of breast milk as the dependent variables. The significance level adopted for all analyses was 5%.

This study complied with the regulatory standards for human research - Resolution No. 466/12 of the National Health Council - and was approved by the Research Ethics Committee of the Universidade Estadual de Montes Claros, under Report No. 1,321,802.

## RESULTS

We evaluated 106 nursing mothers, and 48.1% of them were in the age group 20 to 29 years. Most participants were black/multiracial (81.2%), married (59.4%), and stated that their husband was the head of the family (89.4%). Regarding schooling, 44.3% of the nursing mothers had between 9 and 11 complete years of study, and 51.5% had paid work. As to income, the majority of the sample reported earning less than three minimum wages (91.5%), including benefits, and 28.8% received *Bolsa Família* (a Brazilian welfare program), 4.8% pension, and 1.9% Christian pension funds.

With respect to the nutritional status of the nursing mothers according to BMI, most participants had appropriate weight, both in the pre-gestational assessment (68.8%) and at the last prenatal visit (46.8%). Concerning gestational age, 90.6% of the children were born at term and with adequate weight (3.0 to 3.9 kg) (66%).

Among saturated FA, the palmitic (C16:0), stearic (C18:0), myristic (C14:0), and lauric (C12:0) acids presented higher values, respectively. Out of monounsaturated FA, the oleic (C18:1) and palmitoleic (C16:1) acids had a higher contribution, respectively. The total of essential FA (linoleic and α-linolenic acid) was 14.94% (Table 2).

**Table 1:** Distribution of healthy eating index component scores and servings.

HEI component	Score criteria (Servings recommended by the Guideline for 1,000 calories)	Score
Total fruits ^a^	0 ↔ ≥1.0 serving/1,000 Kcal	0-5
Whole fruits^b^	0 ↔ ≥0.5 serving/1,000 Kcal	0-5
Total vegetables^c^	0 ↔ ≥1.0 serving/1,000 Kcal	0-5
Dark green/orange vegetables and legumes^c^	0 ↔ ≥0.5 serving/1,000 Kcal	0-5
Total grains	0 ↔ ≥2.0 porções/1,000 Kcal	0-5
Whole grains	0 ↔ ≥1.0 serving/1,000 Kcal	0-5
Milk and dairy^d^	0 ↔ ≥1.5 serving/1,000 Kcal	0-10
Meat, eggs, and legumes	0 ↔ ≥1.0 serving/1,000 Kcal	0-10
Oils^e^	0 ↔ ≥0.5 serving/1,000 Kcal	0-10
Saturated fat	≥15 ↔ 10 ↔ ≤7% do TEI	0-8-10
Sodium	≥2.0 ↔ 1.0 ↔ ≤0.7 g/1,000 Kcal	0-8-10
SoFAAS	≥35 ↔ ≤10% TEI	0-20*

Source: adapted from Previdelli et al.^7^

^a^Includes fruits and fresh fruit juice; ^b^excludes fruit juice; ^c^includes legumes only after the component meat, eggs, and legumes reaches the maximum score; ^d^includes milk and soy milk products; ^e^includes mono- and polyunsaturated fats from oleaginous fruits and fish oil; SoFAAS: energy from solid fats, alcohol, and added sugar; *the mean SoFAAS score was calculated by weighting values between 34 and 11 and considering those from 0 to 19.9; TEI: total energy intake.

**Table 2:** Fatty acid composition of breast milk.

Fatty acids	RT	Percentiles						
		Mean±SD	Median	25	75	Minimum	Maximum	p-value*
Saturated	-	52.19±7.62	52.61	48.47	56.97	0.00	66.86	0.066
C8:0 (caprylic acid)	1.79	0.03±0.14	0.00	0.00	0.00	0.00	1.43	0.000
C10:0 (capric acid)	3.08	1.03±0.50	0.98	0.77	1.28	0.00	3.03	0.116
C12:0 (lauric acid)	4.85	5.66±2.36	5.60	4.01	6.89	0.00	11.81	0.726
C14:0 (myristic acid)	6.84	8.12±3.08	7.87	6.21	9.77	0.00	17.16	0.464
C15:0 (pentadecanoic acid)	7.80	0.34±0.15	0.33	0.25	0.41	0.00	1.02	0.068
C16:0 (palmitic acid)	8.90	27.18±4.11	26.89	25.04	29.85	0.00	35.39	0.028
C17:0 (margaric acid)	10.16	0.51±0.43	0.43	0.35	0.52	0.00	3.82	0.000
C18:0 (stearic acid)	10.76	8.17±1.94	8.14	7.15	9.29	0.00	15.13	0.451
C20:0 (arachidic acid)	12.37	0.43±0.75	0.28	0.20	0.42	0.00	6.91	0.000
C22:0 (behenic acid)	13.84	0.73±0.77	0.47	0.39	0.60	0.00	4.54	0.000
Monounsaturated	-	31.92±5.25	32.10	29.18	34.46	0.00	43.02	0.230
C14:1 (myristoleic acid)	7.49	0.24±0.22	0.21	0.12	0.31	0.00	1.33	0.029
C16:1 (palmitoleic acid)	9.72	2.25±0.73	2.31	1.80	2.70	0.00	4.26	0.680
C18:1 (oleic acid)	11.11	27.04±4.88	26.69	24.73	29.51	0.00	38.19	0.390
Polyunsaturated	-	14.94±5.07	15.57	11.48	18.30	0.00	25.03	0.772
C18:2 (linoleic acid)	11.78	13.90±4.74	14.41	10.72	17.01	0.00	23.52	0.726
C18:3 (α-linolenic acid)	12.58	1.04±0.58	1.04	0.60	1.36	0.00	2.71	0.756
Other	-	2.39±1.07	2.39	1.77	2.85	0.00	7.53	0.090

RT: retention time (minutes); *Kolmogorov-Smirnov test indicating that p>0.05, the distribution is normal, and the proper measure of central tendency is the mean; SD: standard deviation.

The HEI component score revealed a higher frequency of maximum scores for the groups total vegetables; dark green and orange vegetables and legumes; meat, eggs, and legumes; oils; and saturated fat. Foods from the groups total fruits, whole fruits, total grains, whole grains, and milk and dairy had a greater frequency of minimum scores (Table 3).

**Table 3:** Healthy eating index component score for nursing mothers.

HEI component	Reference score	Mean±SD	Frequency n (%)	
			Minimum score	Maximum score
Total fruits	0-5	1.42±2.26	76 (71.7%)	30 (28.3%)
Whole fruits	0-5	2.03±2.47	63 (59.4%)	43 (40.6%)
Total vegetables*	0-5	4.43±1.59	12 (11.3%)	94 (88.7%)
Dark green/orange vegetables and legumes*	0-5	4.06±1.97	20 (18.9%)	86 (81.1%)
Total grains	0-5	1.04±2.04	84 (79.2%)	22 (20.8%)
Whole grains	0-5	0.05±0.49	105 (99.1%)	1 (0.9%)
Milk and dairy	0-10	0.57±2.32	100 (94.3%)	6 (5.7%)
Meat, eggs, and legumes	0-10	9.72±1.67	3 (2.8%)	103 (97.2%)
Oils	0-10	9.91±0.97	1 (0.9%)	105 (99.1%)
Saturated fat	0-8-10	7.81±3.08	13 (12.3%)	42 (39.6%)
Sodium	0-8-10	8.53±2.14	5 (4.7%)	48 (45.3%)
SoFAAS	0-20	14.81±6.07	5 (4.7%)	41 (38.7%)
Total score**	0-100	64.36±10.68	-	-

HEI: healthy eating index; *includes legumes only after the component meat, eggs, and legumes reaches the maximum score; **Kolmogorov-Smirnov test (p>0.05) indicating that the distribution is normal and the proper measure of central tendency is the mean; SoFAAS: energy from solid fats, alcohol, and added sugar; SD: standard deviation.

We tested the Pearson’s correlation between the FA profile of breast milk and HEI components and found that the variable total fruits had a negative correlation with the concentration of caprylic, oleic, and linoleic acids, as well as with the whole group of polyunsaturated FA. In the monounsaturated FA group, this correlation was positive. The variable whole fruits showed a negative correlation with the margaric and linoleic acids and the polyunsaturated FA group. We detected a negative correlation between the arachidic acid and the group of dark green and orange vegetables and legumes, and the total grains component was associated negatively with behenic acid and positively with myristic acid and other non-identified FA. The total HEI score was negatively associated only with the margaric and linoleic acids (Table 4).

**Table 4:** Correlation between the fatty acid profile of breast milk and healthy eating index components.

Fatty acids	HEI												
	Total fruits	Whole fruits	Total vegetables	Dark green/orange vegetables and legumes	Total grains	Whole grains	Milk and dairy	Meat, eggs, and legumes	Oils	Saturated fat	Sodium	SoFAAS	HEI total score
C8:0 (caprylic acid)*	-0.230**	-0.044	-0.042	-0.057	0.164	-0.049	-0.124	-0.080	0.049	-0.043	0.041	0.073	-0.023
C16:0 (palmitic acid)*	0.124	0.050	0.024	-0.006	-0.144	0.072	0.060	-0.118	-0.027	-0.100	0.173	-0.039	0.038
C17:0 (margaric acid)*	0.021	-0.224**	-0.053	-0.076	-0.120	0.139	0.079	-0.129	-0.088	0.000	0.034	-0.135	-0.206**
C20:0 (arachidic acid)*	0.046	-0.001	-0.022	-0.193**	0.022	0.030	0.166	0.078	-0.011	-0.149	0.020	0.024	0.008
C22:0 (behenic acid)*	0.151	0.183	0.040	-0.011	-0.218**	-0.092	0.061	0.005	0.053	0.033	0.047	0.055	0.108
C14:1 (myristoleic acid)*	-0.037	-0.086	-0.019	0.089	0.059	-0.037	0.066	0.045	-0.006	0.048	-0.009	-0.013	0.003
C10:0 (capric acid)	0.020	-0.031	-0.055	0.029	0.073	0.060	0.008	0.022	0.047	0.122	-0.057	0.019	0.055
C12:0 (lauric acid)	-0.095	0.011	-0.127	-0.098	0.189	0.012	-0.057	-0.029	0.054	0.106	-0.130	-0.038	-0.047
C14:0 (myristic acid)	-0.042	0.009	-0.109	-0.099	0.263**	-0.018	-0.055	-0.077	0.051	.095	-0.073	-0.031	-0.016
C15:0 (pentadecanoic acid)	0.072	-0.093	0.002	-0.072	-0.093	0.055	0.184	-0.013	0.007	-0.032	0.094	0.027	0.029
C18:0 (stearic acid)	0.060	-0.158	-0.016	-0.056	-0.044	-0.039	0.057	-0.077	-0.033	-0.052	0.038	-0.114	-0.122
C16:1 (palmitoleic acid)	0.021	-0.075	-0.025	0.019	-0.103	0.148	-0.065	0.114	-0.046	-0.179	0.047	-0.020	-0.080
C18:1 (oleic acid)	0.364**	0.111	0.033	0.090	-0.182	0.071	0.030	0.068	0.018	-0.071	-0.068	0.065	0.114
C18:2 (linoleic acid)	-0.320**	-0.296**	-0.016	-0.055	0.075	-0.072	0.008	0.061	-0.078	-0.188	-0.114	0.012	-0.204**
C18:3 (α-linolenic acid)	-0.020	-0.056	0.012	0.034	0.014	-0.009	0.098	0.062	-0.023	-0.027	0.006	0.113	0.080
Other	-0.099	-0.020	0.151	0.176	0.315**	-0.069	-0.028	0.088	0.010	0.159	0.081	-0.020	0.145
Saturated	0.057	-0.021	-0.077	-0.116	0.098	0.008	0.008	-0.132	0.030	0.068	0.011	-0.058	-0.034
Monounsaturated	0.322**	0.086	0.060	0.127	-0.119	0.071	0.015	0.099	0.012	-0.055	-0.039	0.049	0.125
Polyunsaturated	-0.302**	-0.283**	-0.014	-0.048	0.072	-0.068	0.019	0.064	-0.076	-0.179	-0.106	0.024	-0.182

HEI: healthy eating index; SoFAAS: energy from solid fats, alcohol, and added sugar; *Spearman’s correlation; **the correlation is significant at the p<0.05 level.

In the multiple linear regression analysis that assessed the relationship between the maternal diet quality and the FA composition of breast milk, the HEI variable total fruits showed a negative association with the percentage of polyunsaturated FA and linoleic acid. For the monounsaturated oleic and behenic acids, the relationship was positive. Some non-identified FA were associated with the components total vegetables and total grains, and the myristic acid was related to the group of total grains, as shown in Table 5. The HEI component total fruits showed an inverse correlation with the polyunsaturated FA group and linoleic acid, and a positive relationship with the monounsaturated FA group, oleic acid, and behenic acid. The components total vegetables and total grains had a positive correlation with other non-identified FA, and total grains also presented a positive association with the myristic acid.

**Table 5:** Final multiple linear regression model of the relationship between the fatty acid profile of breast milk and healthy eating index components.

Dependent variable	HEI component	Raw coefficient	Standardized coefficient	95%CI		p-value
				LL	UL	
Polyunsaturated	(Constant)	15.901	-0.302	14.797	17.005	0.000
	Total fruits	-0.676		-1.091	-0.261	0.002
Monounsaturated	(Constant)	30.862	0.322	29.727	31.997	0.000
	Total fruits	0.747		0.320	1.173	0.001
Other	(Constant)	1.549	0.216 0.355	0.943	2.155	0.000
	Total vegetables	0.145		0.022	0.269	0.022
	Total grains	0.186		0.090	0.283	0.000
C18:2 (linoleic acid)	(Constant)	16.675	-0.303	14.295	19.055	0.000
	Total fruits	-0.636		-1.021	-0.251	0.001
C18:1 (oleic acid)	(Constant)	25.934	0.364	24.896	26.972	0.000
	Total fruits	0.785		0.395	1.175	0.000
C22:0 (behenic acid)	(Constant)	0.656	0.281	0.469	0.843	0.000
	Total fruits	0.096		0.033	0.159	0.003
C14:0 (myristic acid)	(Constant)	7.706	0.263	7.059	8.352	0.000
	Total grains	0.398		0.114	0.682	0.006

HEI: healthy eating index; 95%CI: 95% confidence interval; LL: lower limit; UL: upper limit.

## DISCUSSION

The proper eating habits of nursing mothers are important, as they directly affect the quality of breast milk and can lead to consequences for the infant.^9^ Thus, this study aimed to evaluate the relationship between the maternal diet quality and the FA composition of breast milk in the first trimester of lactation.

With respect to the lipid profile, the mean percentage of saturated and monounsaturated FA was lower than the findings from Santos et al.,^18^ who evaluated the serum FA profile of adolescent nursing mothers in Rio de Janeiro and found 36% of saturated FA and 19.4% of monounsaturated FA. However, this same study identified a higher mean percentage of polyunsaturated FA than that found in the present research. The concentration of palmitic, myristic, lauric, oleic, palmitoleic, linoleic, and α-linolenic acids was close to the values reported in the literature.^19^

Considering the reference score for each HEI component, this investigation revealed that most nursing mothers assessed presented minimum scores for the intake of total fruits, whole fruits, total grains, whole grains, and milk and dairy, going against the recommendations from the Dietary Guidelines Advisory Committee^20^ and the Dietary Guidelines for the Brazilian Population,^15^ which propose the consumption of two or three servings of milk and dairy products and three servings of fruit per day in the postpartum period.

The results of this study agree with findings from George et al.,^21^ who identified that the consumption of grains and fruits decreases, and the sugar intake increases during the postpartum period. Durham et al.^22^ also reported that the consumption of fruits, grains, and dairy products is low. In Brazil, other works investigating the same population group also showed that diets were restricted as to the food variety, with low intake of fruits and vegetables.^9^ ^,^ ^23^

Regarding the variables related to the HEI of nursing mothers, the final multiple linear regression model revealed that the components total fruits, total vegetables, and total grains were associated with poly- and monounsaturated FA; linoleic, oleic, behenic, and myristic acids; and other non-identified FA.

The total fruit component had an inversely proportional relationship with the polyunsaturated FA group and the linoleic acid, that is, the higher fruit intake, the lower the concentration of polyunsaturated FA. Valenzuela and Nieto^24^ corroborate this information, since the main sources of polyunsaturated FA, such as linoleic acid, are fishery products, including fishes, crustaceans, mollusks, amphibians, chelonians, and mammals from fresh or salt water, with tuna, sardine, and salmon being the primary sources of these nutrients.

Foods like oleaginous fruits, seeds, vegetables, egg yolk, octopus, and ruminant meat are also relevant sources of omega-3.^25^ A study performed by Leite et al.^26^ in female rats showed that linseed is a good source of protein and lipids, providing adequate growth to the pups during lactation; however, they emphasized the importance of further studies to assess the transfer of omega-3 FA from this seed to breast milk.

Valenzuela and Nieto^24^ underlined the importance of a diet rich in vegetables and fishery products, especially seafood, which is a nutritional source of polyunsaturated FA of the omega-3 and omega-6 series, but less consumed by the general population, including pregnant women and nursing mothers.

The total fruit component had a directly proportional relationship with the monounsaturated FA group and the oleic acid, that is, the higher fruit intake, the greater the concentration of monounsaturated FA in the breast milk. According to Nascimento et al.,^27^ oleaginous fruits such as the açaí berry, olive, and avocado are rich in monounsaturated FA. Thus, the consumption of oleaginous fruits during pregnancy and lactation would also increase the lipid profile of monounsaturated FA, such as oleic acid, in breast milk.

We could identify a directly proportional relationship between the total fruit component and the concentration of behenic FA, and between total grains and the myristic acid. Saturated FA are considered an energy source or substrates to synthesize intermediate compounds.^28^ Part of the content of these FA found in breast milk can be synthesized *de novo* in the mammary gland, through glucose, and its first result is the formation of saturated FA with 10 to 14 carbon atoms,^3^ which explains the positive relationship between the behenic FA and the consumption of total fruits. The generation of saturated FA increases when the maternal diet consists of low levels of lipids and high levels of grains, as shown in a study conducted by Glew et al.^29^

These findings demonstrate that the lipid modulation of milk does not result from isolated effects, but from several factors intrinsic and extrinsic to the nursing mother. Evidence shows that the maternal diet is the main modulation factor of the lipid composition of breast milk;^3^ however, we emphasize that few studies have evaluated the relationship between the FA profile of breast milk and the consumption of these nutrients by nursing mothers, which suggests the need for a careful assessment of their eating habits.

Few investigations have compared the diet quality of nursing mothers using HEI with the FA profile of breast milk, which restricts the contrast of results. This study revealed the inverse relationship between the consumption of total fruits and the profile of polyunsaturated FA, and the direct association of the consumption of total fruits and total grains with monounsaturated and saturated FA, indicating that the diet quality influences the composition of breast milk. Therefore, nursing mothers should receive nutritional guidance encouraging the adoption of healthier eating habits, including the reduction of foods rich in saturated and trans fats and the increase in the consumption of mono- and polyunsaturated fats.

The main objective of this study was to evaluate the relationship between the maternal diet quality and the FA composition of breast milk in the first trimester of lactation, but some limitations must be taken into account, and the results herein should be interpreted with caution. First, we did not explore all possible factors that could interfere in the FA profile of breast milk, such as smoking and alcoholism. Another limitation of this study concerns the dietary data collection instrument (recall of usual intake), which relies on the memory of the interviewee and the interviewer’s ability to communicate with the participants, in addition to the difficulty in estimating the size of the servings. Nonetheless, the researchers adopted some procedures to minimize measurement bias during data collection, such as previous and frequent training of the interviewers in the correct approach and standardization of utensils to facilitate the identification of household measures.

Given the importance of essential FA, prenatal visits, particularly in the last trimester of pregnancy, and pediatric appointments should investigate the maternal diet, instructing them as to the dietary consumption of foods rich in essential FA, such as the docosahexaenoic acid, since WHO recommends an intake between 200 and 600 mg of this acid by pregnant and lactating women, either through source foods, such as fish from cold waters, or by supplementation.
